# The cholinergic anti-inflammatory pathway inhibits inflammation without lymphocyte relay

**DOI:** 10.3389/fnins.2023.1125492

**Published:** 2023-04-14

**Authors:** Thomas Simon, Joseph Kirk, Nikola Dolezalova, Mélanie Guyot, Clara Panzolini, Alexandre Bondue, Julien Lavergne, Sandrine Hugues, Nicolas Hypolite, Kourosh Saeb-Parsy, Justin Perkins, Eric Macia, Arun Sridhar, Margriet J. Vervoordeldonk, Nicolas Glaichenhaus, Matteo Donegá, Philippe Blancou

**Affiliations:** ^1^Université Côte d’Azur, CNRS, Molecular and Cellular Pharmacology Institute, Valbonne, France; ^2^The Royal Veterinary College, Hatfield, United Kingdom; ^3^Department of Surgery, University of Cambridge and NIHR Cambridge Biomedical Research Centre, Cambridge, United Kingdom; ^4^E-PHY-SCIENCE, Valbonne, France; ^5^Galvani Bioelectronics, Translational Sciences, Stevenage, United Kingdom

**Keywords:** splenic nerve stimulation, cholinergic anti-inflammatory pathway, CD4+ T lymphocytes, myeloid cells, beta2 adrenergic receptor

## Abstract

The magnitude of innate inflammatory immune responses is dependent on interactions between peripheral neural and immune cells. In particular, a cholinergic anti-inflammatory pathway (CAP) has been identified in the spleen whereby noradrenaline (NA) released by splenic nerves binds to ß2-adrenergic receptors (β2-AR) on CD4^+^ T cells which, in turn, release acetylcholine (ACh). The binding of ACh to α7 acetylcholine receptors (α7-AChR) expressed by splenic macrophages inhibits the production of inflammatory cytokines, including tumor necrosis factor (TNF). However, the role of ACh-secreting CD4^+^ T-cells in the CAP is still controversial and largely based on the absence of this anti-inflammatory pathway in mice lacking T-cells (nude, FoxN1^−/−^). Using four conscious, non-lymphopenic transgenic mouse models, we found that, rather than acting on CD4^+^ T-cells, NA released by splenic nerve terminals acts directly onto β2-AR on splenic myeloid cells to exert this anti-inflammatory effect. We also show that, while larger doses of LPS are needed to trigger CAP in nude mouse strain compared to other strains, TNF production can be inhibited in these animals lacking CD4^+^ T-cell by stimulating either the vagus or the splenic nerve. We demonstrate that CD4+ T-cells are dispensable for the CAP after antibody-mediated CD4+ T-cell depletion in wild type mice. Furthermore, we found that NA-mediated inhibition of *in vitro* LPS-induced TNF secretion by human or porcine splenocytes does not require α7-AChR signaling. Altogether our data demonstrate that activation of the CAP by stimulation of vagus or splenic nerves in mice is mainly mediated by direct binding of NA to β2-AR on splenic macrophages, and suggest that the same mechanism is at play in larger species.

## Introduction

The seminal observations by Borovikova et al. that LPS-induced pro-inflammatory cytokines secretion is inhibited both by acetylcholine receptor (AChR) agonists *in vitro* and by vagus nerve electrical stimulation (VNS) *in vivo* (Borovikova et al., 2000) led to the concept that parasympathetic nerves control inflammation in a cholinergic-dependent manner. This pathway was later called the cholinergic anti-inflammatory pathway (CAP) (Tracey, 2002). Further studies showed that the splenic nerve is a necessary relay for CAP (Rosas-Ballina et al., 2008; Peña et al., 2011), and that the release of acetylcholine by CD4^+^ T-cells (Rosas-Ballina et al., 2011) by β2 adrenergic receptors (β2-AR) activation (Vida et al., 2011b) is required. The fact that the CAP was not observed in α7-AChR knock-out animals (Wang et al., 2003; Huston et al., 2006; Vida et al., 2011a) and that nicotine exerts anti-inflammatory effects on macrophages *via* α7-AChR (Borovikova et al., 2000; Wang et al., 2003; Uni et al., 2020) led to the notion that α7-AChR expressed on macrophages is the main anti-inflammatory mediator of vagus nerve cholinergic anti-inflammatory output (Gallowitsch-Puerta and Pavlov, 2007; Jonge and Ulloa, 2009; Andersson and Tracey, 2012; Olofsson et al., 2012). However, α7-AChR was also demonstrated to play a role in the neural connection between the vagus and SpN (Vida et al., 2011a), thus questioning the role of α7-AChR on macrophages in the CAP.

The connection between the vagus and the splenic nerve is also controversial (Martelli et al., 2014a). Some authors found that the vagus nerve projects to the spleen either directly (Chen et al., 1996; Buijs et al., 2008; Cailotto et al., 2012; Gautron et al., 2013; Ting et al., 2017) or through a ganglionic relay (Vida et al., 2011a), while others could not demonstrate an anatomical or an electophysiological connection between the vagus and the splenic nerve (Bratton et al., 2012; Martelli et al., 2014b). We have recently shown that splenic innervation in mice is more complex than initially thought with three catecholaminergic branches projecting to the spleen in mice including one branch containing cholinergic fibers (Guyot et al., 2019). Whether and how these splenic nerve branches are connected to the vagus nerve is still unclear.

To clarify these issues, we have conducted an electrophysiological study including all branches of nerve projecting to the spleen and we investigated the CAP in conscious, non-lymphopenic transgenic mouse models. We also have tested our hypothesis *in vitro* in Pigs and Humans.

## Materials and methods

### Animals

All animal studies were ethically reviewed and carried out in accordance with Animals (Scientific Procedures) Act 1986 and the Galvani Policy on the Care, Welfare and Treatment of Animals. Protocols were approved by the Comité Institutionnel d’Éthique Pour l’Animal de Laboratoire (CIEPAL) and the Royal Veterinary College Animal Welfare for mice and pigs, respectively, as well as the Ethical Review Board and the Galvani Animal and Scientific Review Committee for both species.

C57BL/6 and Balb/c.FoxN1^−/−^ were purchased from Charles River (France). CD4:Cre, LysM:Cre, ADRB2^−/−^, RAG-1^−/−^ and ChAT^LoxP/LoxP^ mice were purchased from The Jackson Laboratory. ADRB2^LoxP/LoxP^, was kindly provided by Gerard Karsenty (Hinoi et al., 2008). ADRB2^−/−^ was backcrossed on the C57BL/6 background for at least 10 generations. All experiments were performed with female 8–16 weeks old mice. Mice were housed on a 12 h light/dark cycle (lights on/off at 7 am/7 pm) with food *ad libitum*.

All pigs were housed and transported under conditions specified in the UK’s Animal Welfare Act 2006 and the Welfare of Farm Animals (England) Regulations 2007. Female farm pigs (Large white/British landrace cross, body weight 62–81 kg, aged 15–17 weeks) were sourced from a specific pathogen free commercial pig farm (indoor housing) and acclimatised at the research facility for a minimum of 7 days prior to terminal experiment. Pigs were group housed, given *ad libitum* access to water, fed a commercial pelleted sow and weaner diet and straw bedded with environmental enrichment during the acclimatisation period. Food, but not water, was withheld 12 h prior to surgery.

### Electrostimulation of the splenic and vagus nerves in mice

Mice were pre-medicated with buprenorphine (100 μg/kg, i.p.) 30 min before surgery and anesthetized with isoflurane (2% v/v) for the duration of the surgery. For splenic nerve implantation, 1 mm length 100 μm-sling bipolar micro-cuff electrodes (CorTec) were implanted onto the arterial main splenic nerve. For vagus nerve implantation, 2 mm length 200 μm-tunnel bipolar micro-cuff electrodes (CorTec) were implanted onto the left vagus nerve.

Seven days following surgery, implanted mice were injected i.p. with a different dose of LPS and electrostimulation was applied using a PlexStim V2.3 (Plexon) starting at −10, 0 and +20 min relative to LPS injection. Sera was collected at 90 min after LPS injection and assessed for TNF levels. Controls consist of fully Cortec implanted mice, which did not receive electrical stimulation (NS). Electrostimulation were rectangular charged-balanced biphasic pulses with 650 μA pulse amplitude, 100 μs pulse width (positive and negative) at 10 Hz frequency for 2 min (S). Ninety minutes after LPS injection, retro-orbital blood sampling was performed under isofluorane anesthesia.

### Recordings of compound action potentials

Mice were pre-medicated with buprenorphine (100 μg/kg, i.p.) 30 min before surgery and anesthetized with isoflurane (2% v/v) for the duration of the experiment. A hook electrode was placed onto the vagus nerve for stimulation with Master-8 (A.M.P.I.) and rectangular charged-balanced biphasic pulses with different pulse amplitudes, 100 μs pulse width (positive and negative) at 5 Hz frequency were applied. A platinum-iridium recording electrodes (Phymep) were placed onto the different splenic nerve branches (apical, middle and main), for CAP recording using a wireless recording system (W8, Multi-Channel Systems) with a 10 kHz sampling rates. Data were analysed after applying a 10-1 kHz filter and averaging 100 sweeps of individual stimuli. Ground/Reference wires were placed into the nearby tissue. All recordings were performed in a Faraday cage.

### *In vivo* CD4+ T cells depletion

Depletion was performed by two i.p. injection at day −2 and −1 of 200 μg of purified monoclonal rat anti-mouse CD4 Ab (Clone GK1.5, Bioxcell). Control (nondepleted) mice were treated similarly with rat IgG2b isotype (Clone LTF-2, Bioxcell). CD4+ T cell depletion in the spleen was assessed by flow cytometry.

### Porcine splenocyte preparation

General anaesthesia was induced as above. After aseptic surgical preparation, the spleen was accessed *via* the left lateral abdomen. The major vessels (splenic, short gastric, and gastroepiploic arteries and veins) were sequentially ligated, and the vessels transected. The omentum was incised and the spleen removed. Animals were then subjected to other procedures not related to this manuscript and then euthanized by barbiturate overdose.

Spleens were cut into four sections, 5 g of tissue were sampled from the middle of each section, rinsed in cold PBS (10,204,733, Fisher Scientific) and passed through a metal strainer under gentle manual pressure using a 50 ml syringe plunger. Cell suspension was transferred to six 50 ml conical centrifuge tubes (E1450-0200, Starlab) and centrifuged for 10 min, 300 ×*g*, at 4°C. Supernatant was discarded and cells resuspended in 20 ml cold PBS, then mixed with 30 ml erythrocyte lysis buffer (8.29 g ammonium chloride (21236.267, VWR International Ltd., Lutterworth, United Kingdom), 1 g potassium hydrogen carbonate (237,205, Sigma-Aldrich Ltd., St. Louis, United States), 37 mg EDTA disodium salt (ED2SS, Sigma-Aldrich) dissolved in 1 L DI water and filter sterilised) and left to stand for 10 min, mixing by inversion half way through. Centrifugation was repeated and supernatant discarded, erythrocyte lysis was repeated. Leukocyte pellets were resuspended in 20 ml cold PBS and centrifuged for 5 min, 300 ×*g*, at 4°C. Cells were resuspended in 20 ml cold PBS and passed through 70 μm cell strainers (352,350, Scientific Laboratory Supplies Ltd., Nottingham, United Kingdom) into fresh tubes to remove debris, pooled, then centrifuged for 5 min as previously. Supernatant was discarded and cells resuspended in complete culture medium and counted by trypan blue exclusion (15,250,061, Fisher) using counting chambers (BVS100, Immune Systems Ltd., Paignton, United Kingdom).

Cells were resuspended in medium [RPMI 1640 (72400-021, Fisher Scientific), supplemented with 10% Foetal Bovine Serum (FBS) (11,550,356, Fisher Scientific) and 1% Penicillin–Streptomycin (11,548,876, Fisher Scientific)] and plated at 5 × 10^5^/mL, in either 48- or 12-well flat bottomed culture plates as necessary (CC7682-7548 & CC7682-7512 respectively, Starlab). Following plating, cells were placed in an incubator (37°c, 5% CO_2_) to acclimatise whilst treatments were prepared.

### Human splenocyte preparation

Human spleens were retrieved using one of the following routes: excision of spleen samples from deceased human organ donors under ethical approval (REC Ref: 15/EE/0152) and after obtaining informed consent for use in research from the donor family, or attached to pancreases intended for transplantation and subsequently declined by multiple transplant centres (under ethical approval REC Ref: 16/EE/0227). Total of 16 deceased human organ donors were used for this study: 56% males, 44% females, average age 47.1 ± 13.2 (range 20–62); spleens were processed on average 32.1 ± 18.5 h after death (range 1.5–67 h). Peripheral blood was obtained through NHS Blood and Transplant Non-Clinical Issue (NCI) and processed within 14 days of collection.

Spleen was cut into small pieces, immersed in PBS + 2% FBS and dissociated into single cell suspension in C tubes using a pre-set programme on the gentleMACS Dissociator (Miltenyi Biotec). Cell suspension was immediately filtered through a 70 μm cell strainer. Peripheral blood was diluted in 1:1 ratio with PBS + 2% FBS and filtered through a 70 μm cell strainer. 20 ml of spleen or peripheral blood suspension was carefully layered on top of 15 ml Lymphoprep (Stemcell Technologies) and centrifuged at 800 g for 25 min at 20°C with the brake off. Mononuclear cell fraction was collected at the interface and washed with PBS + 2% FBS to remove residual Lymphoprep.

Cells were counted and resuspended in culture medium (RPMI +10% FBS + 1% Penicillin/Streptomycin) at 1 × 106 cells/mL, plated in 48-well plate (500 μl per well) and kept at 37°C and 5% CO_2_ in humidified atmosphere until the start of the assay.

### Incubation protocols for human and pig splenocytes

LPS (Sigma Life Sciences, cat. no. L4391) and L-Norepinephrine hydrochloride (Sigma Life Sciences, cat. no. 74480) were reconstituted in saline at 1 mg/ml and 10 mg/ml, respectively, and aliquots stored at −20°C. α-Bungarotoxin (cat. no. 2133) was reconstituted with ddH_2_O at 1 mM, Methyllycaconitine citrate (cat. no. 1029), Hexamethonium bromide (cat. no. 4111), Phentolamine mesylate (cat. no. 6431) and (S)-(−)-Propranolol hydrochloride (cat. no. 0834, all from Tocris Bioscience) were reconstituted with ddH_2_O at 100 mM. All compounds were stored at −20°C then thawed at room temperature and added to cells 30 min before LPS and/or NA at final concentrations of 0.15–15 μg/ml (α-Bungarotoxin), 0.1–100 μM (Methyllycaconitine, Hexamethonium & Phentolamine), and 0.003–300 μM (Propranolol). Aliquots of NA and LPS were thawed at room temperature, and LPS sonicated for 5 min. Compounds were diluted in culture medium as required, vortexing for 10 s between each serial dilution for dose–response experiments.

In NA dose–response experiments, plates with splenocytes were removed from the incubator and 10.3 μl of each NA concentration was added to duplicate wells, giving final concentrations between 80 μM and 8 × 10^−5^ μM NA. 5 μl of 10 μg/ml LPS was added to appropriate wells, to give a final concentration of 100 ng/ml LPS. Duplicate negative control (medium only) and positive control (LPS only) treatments were included. Plates were returned to the incubator for 3 h.

In receptor antagonism experiments, plates with splenocytes were removed from the incubator and receptor antagonists were added to duplicate wells as appropriate. Plates were returned to the incubator for 30 min pre-treatment. Plates were again removed from the incubator and 41 μl of NA and 20 μl of LPS were added to duplicate wells as appropriate, giving final concentrations of 8 μM NA and 100 ng/ml LPS. Plates were returned to the incubator for 3 h.

Following incubation, contents of wells were transferred by pipette to 1.5 ml microcentrifuge tubes (E1415-2231, Starlab) and centrifuged at 2,000 ×*g* for 5 min. The conditioned medium (CM) from each tube was collected, aliquoted, and immediately frozen on dry ice, before transfer to −80°C storage.

### Magnetic separation of human splenocyte populations

CD4 depletion was performed using human CD4 MicroBeads (Miltenyi Biotec, cat. no. 130-045-101; >99% depletion efficiency) according to manufacturer’s instructions. Magnetic separation was performed using the autoMACS Pro Separator (Miltenyi Biotec). Purity of the isolated subsets was checked using flow cytometry after staining with a cocktail of antibodies containing CD14 PE-Vio770 (Miltenyi Biotec, cat. no. 130-098-074), CD3 APC (BioLegend, cat. no. 3004399) and CD4 FITC (Miltenyi Biotec, cat. no 130-114-722). Absolute number of CD14^+^ cells used for subsequent incubations was determined using BD Trucount Tubes (BD, cat. no. 340334) and no-wash staining procedure with the above antibody cocktail.

### Flow cytometry

Human cells were resuspended in a staining buffer (PBS + 0.1% bovine serum albumin +0.1% sodium azide) and treated with human FcR blocking agent (Miltenyi Biotec, cat. no. 130–059-901) for 10 min at 4°C prior to staining. Samples were stained with primary Anti-beta2-adrenergic receptor Ab (Abcam, cat. no. ab61778) for 30 min at 4°C. After three washing steps with a staining buffer, Donkey anti-rabbit IgG H + L (AlexaFluor488) (Abcam, cat. no. ab150073) was added, together with the viability stain 7-AAD (BioLegend, cat. no. 420404) and the remaining conjugated antibodies [CD19 PE (BioLegend, cat. no. 363004), CD14 PE-Vio770 (Miltenyi Biotec, cat. no. 130–098-074) and CD3 APC (BioLegend, cat. no. 3004399)] for 20 min 4°C protected from light. Samples were washed three times prior to analysis on a flow cytometer. Along with the samples, appropriate controls were prepared, including unstained control and compensation controls using BD™ CompBeads Anti-Mouse Ig, κ/Negative Control (BD Bioscience, cat. no. 552843). Secondary only controls were prepared by omitting the primary Anti-beta2-adrenergic receptor Ab staining step. The analysis of the stained samples was done using a BD FACSCantoTM II flow cytometer and computer equipped with the BD FACSDivaTM software (BD Biosciences, version 6.1.3). Data was analysed by the FlowJo software (version 10.6.1).

### ELISAs

For mice, TNF levels were measured by ELISA (Mouse TNF-alpha DuoSet, R&D Systems) following manufacturer instructions and normalized to sham stimulated animals. The limit of detection was 15 pg./ml. Porcine samples were analysed by ELISA for quantification of TNF using Porcine DuoSet ELISA kit (Bio-Techne Ltd., cat. no. DY690B). Plates were analysed using the Infinite^®^ 200 PRO spectrophotometer and iControl software (Tecan Group Ltd.).

Human samples were analysed for concentration of TNF using TNF alpha Human ELISA Kit (Invitrogen, cat. no. KHC3011) and for Ach using the Universal Acetylcholine ELISA Kit (Colorimetric) (Bio-Techne Ltd., cat. no. NBP2-66389) according to manufacturer’s instructions. Plates were analysed using the BMG FLUOstar Optima plate reader (BMG Labtech).

### Chemicals

Methyllycaconitine citrate (cat. no. 1029, reconstituted with ddH_2_O), Phentolamine mesylate (cat. no. 6431, reconstituted with ddH_2_O) and (S)-(−)-Propranolol hydrochloride (cat. no. 0834, reconstituted with ddH_2_O) were purchased from Tocris Bioscience. LPS (cat. no. L4391, reconstituted in saline at 1 mg/ml) and L-Norepinephrine hydrochloride (cat. no. 74480, reconstituted in saline at 10 mg/ml) were purchased from Sigma Life Sciences.

### Statistics

**Figure 1:**
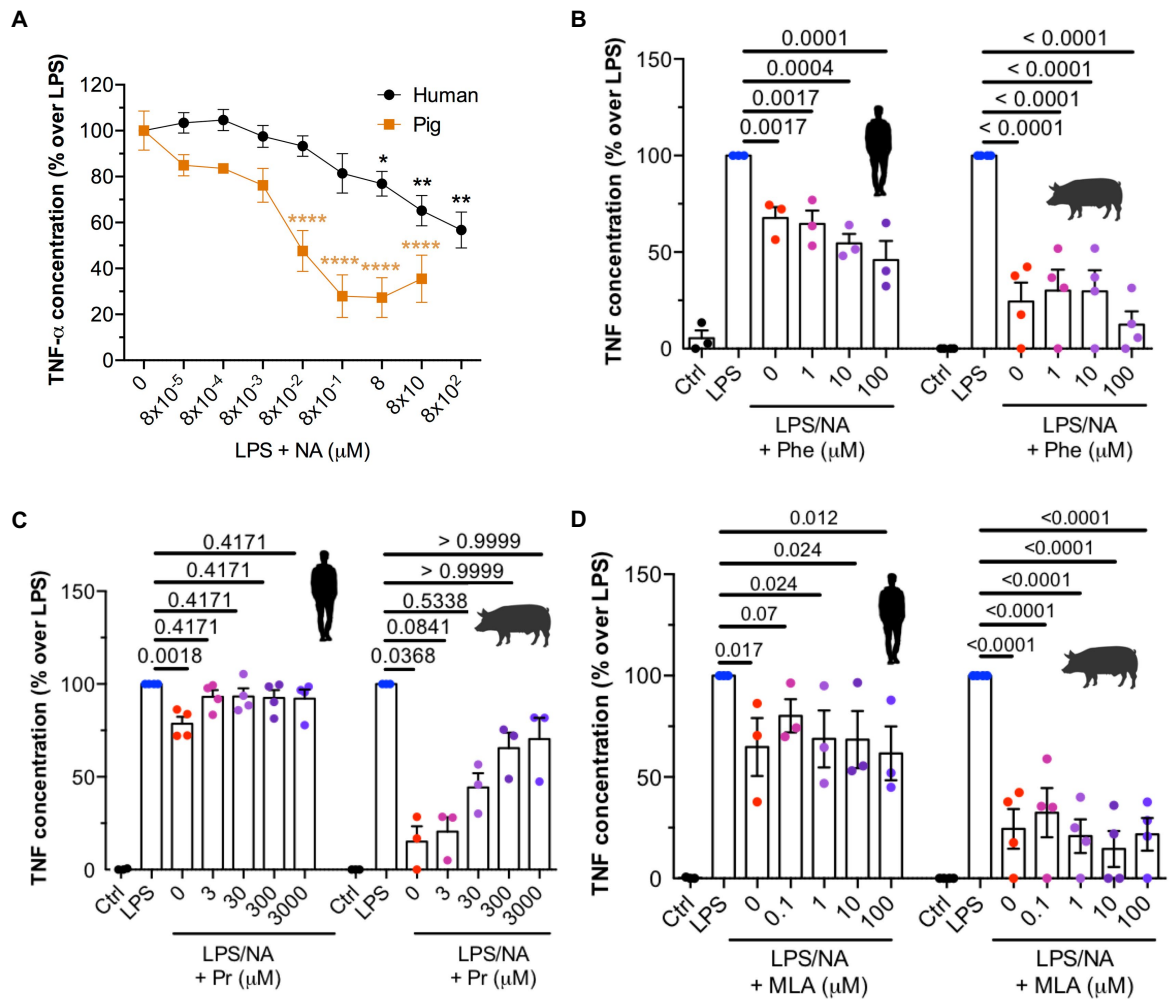
Efferent vagus nerve is connected to the splenic nerves. **(A)** Mice were anesthetized, a hook electrode was placed on the left vagus nerve and a platinum-iridium recording electrodes were placed onto the apical, middle or main branches of the splenic nerve for evoked compound action potential recording. Representative (of 4 mice) **(B)** and maximal amplitudes **(C)** of evoked compound action potential recordings (average of 100 sweeps) in the indicated tissues after vagus nerve stimulation (VNS). Stimulation artefacts are indicated by arrow. **(C)** Mean ± SEM. Representative (of 3 mice). **(D,E)** CAP recordings in middle arterial splenic nerve after either coeliac superior mesenteric ganglion (CG/SMG) cauterization **(D)** or α7-AchR specific antagonist methyllycaconitine (MLA) i.v. administration **(E)**.

**Figure 2:**
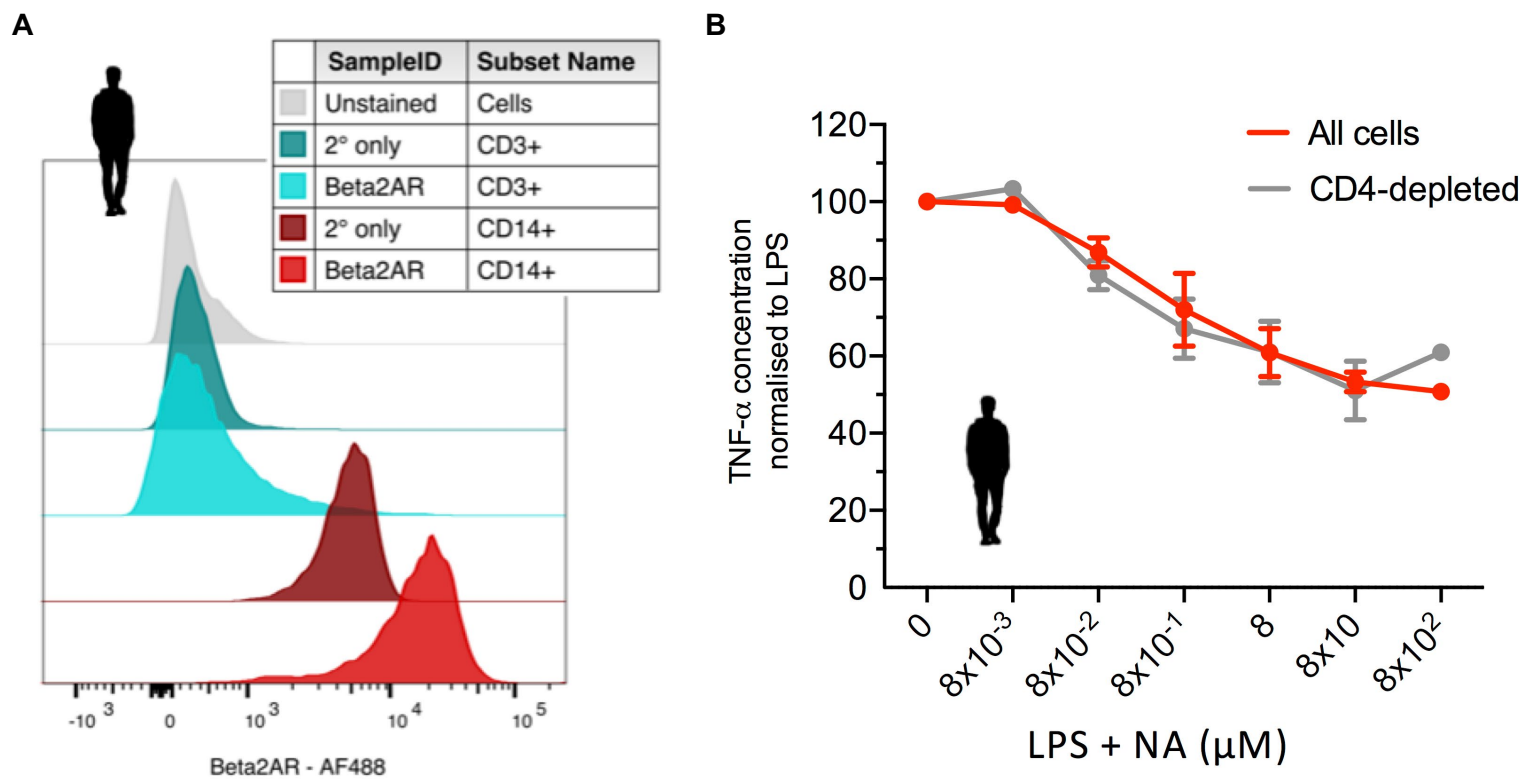
Cholinergic anti-inflammatory pathway is independent of CD4+ T-cells in mice. Wild type **(A)**, CD4:Cre ADRB2fl/fl **(B)**, CD4:Cre CHATfl/fl **(C)** mice and LysM:Cre ADRB2^fl/fl^ **(F)** mice carrying (fl/fl) or not (LoxP/LoxP) the Cre transgene and RAG-1^−/−^ **(E)** mice and control littermates were implanted onto the vagus or main arterial splenic nerve with a micro-cuff electrode 1 week before electrostimulation was applied. **(D)** Wild type mice were i.p. injected with CD4 depleting antibody or isotype control on day −3 and − 1 relative to electrostimulation. One week after surgery, LPS (5 mg/kg) was injected and electrical stimulation was applied or not (650 μA, 10 Hz, 2 min duration, −20, 0 and + 10 relative to LPS injection) to the vagus (upper panels) or main arterial splenic nerve (lower panels) in freely moving animals. Data show serum TNF levels in individual mice ±SEM of 2–5 experiments in non-electrically stimulated (NS, blue) and electrically stimulated (S, red) mice. **(A–F)** Mann–Whitney were performed. *p < 0.05; **p < 0.01; ***p < 0.001; ****p < 0.0001.

**Figure 3:**
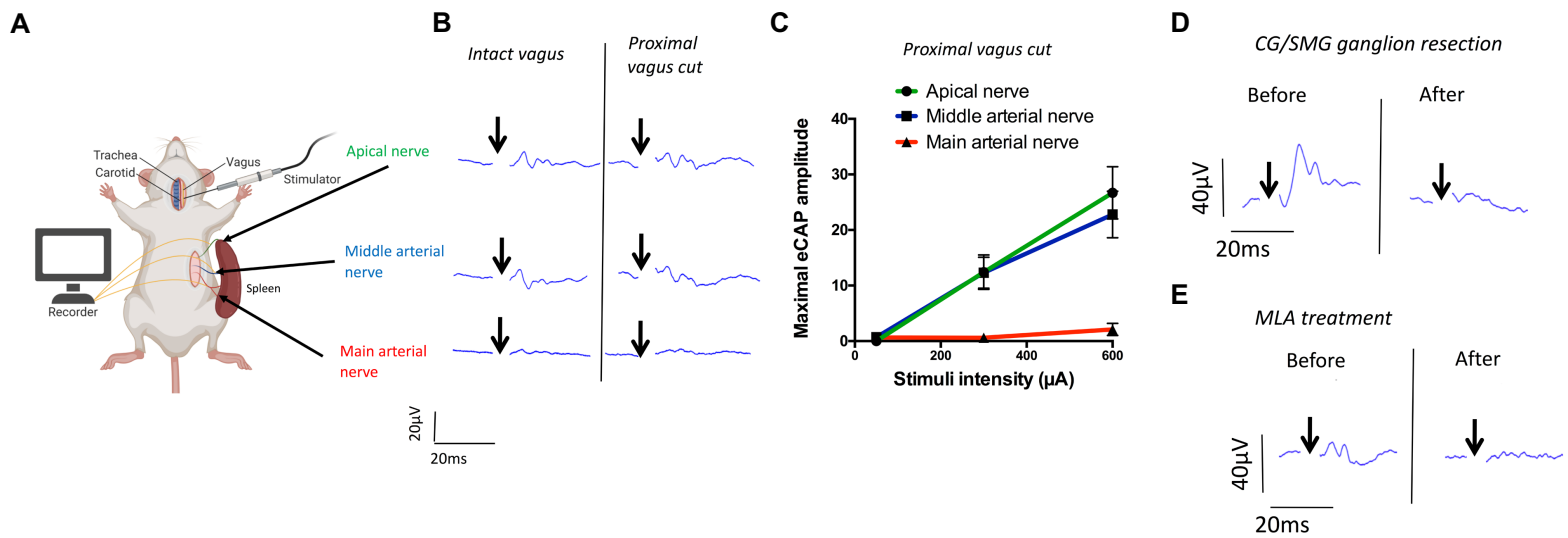
Cholinergic anti-inflammatory pathway in nude (FoxN1^−/−^) mice. Nude mice were implanted onto the vagus **(A)** or arterial splenic **(A–C)** nerve with a micro-cuff electrode 1 week before electrostimulation was applied. **(C)** One, two or five millions CD4^+^ T-cell (1 M, 2 M, 5 M) or PBS (0 M) were transferred on day −1 relative to electrostimulation. One week after surgery, LPS was injected and electrical stimulation was applied or not (650 μA, 10 Hz, 2 min duration, −20, 0 and +10 relative to LPS injection) in freely moving animals. Data show serum TNF levels in individual mice ±SEM of 2–5 experiments (*n* ≥ 5/group) in non-electrically stimulated (NS, blue) and electrically stimulated (S, red) mice. **(A,C)** Mann–Whitney and **(B)** Kruskal–Wallis followed by Dunn’s *post-hoc* test were performed.

Normality of sample distribution was assessed using the D’Agostino and Pearson omnibus normality test. Comparison between unstimulated *vs* stimulated condition were performed using either unpaired Mann–Whitney or unpaired t-test for data that did not or did pass normality test, respectively. For comparison between the three groups in Figures 1B,C, statistical significance was assessed using One-way ANOVA followed by Holm-Sidak *post-hoc* test. For comparison between the groups in Figures 2, 3, statistical significance was assessed using Kruskal–Wallis test followed by Dunn’s *post-hoc* test. All statistical analysis were performed using GraphPad Prism v.6. All statistical analysis were performed using GraphPad Prism v.6. For Figure 2 Friedman test were applied except for panel h where a two-way ANOVA test corrected by Sidak was applied.

## Results

**Figure 4:**
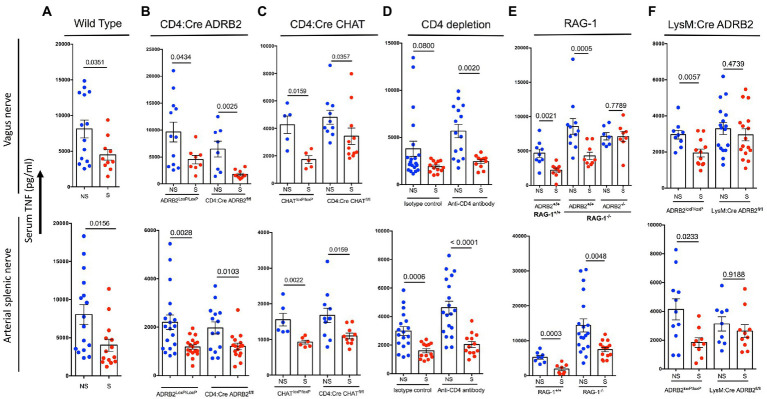
NA promotes TNF suppression *via* β_2_-AR and is independent of α7-AChR in humans and pigs. **(A)** Dose dependent change of LPS-induced TNF caused by treatment of human (in black) and pig (in grey) splenocytes with different concentrations of NA (8 × 10^−5^ – 8 × 10^2^ μM). Data are expressed as % over LPS control (*n* ≥ 5). **(B–D)** Quantification of TNF in human and pig splenocytes in medium only (Ctrl), LPS (100 ng/ml), LPS (100 ng/ml) + NA (8 μM) and LPS (100 ng/ml) + NA (8 μM) in the presence of various concentrations of α-AR (Phentolamine, Phe), β-AR (Propranolol, Pr) or α7-AChR (methyllycaconitine, MLA) receptor antagonists. Data are expressed as % over LPS control (*n* ≥ 3). All data are expressed as mean ± SEM. For statistical analysis a Kruskal–Wallis followed by Dunn’s *post-hoc* test were performed.

To clarify the anatomical connections between the vagus nerve and the spleen, we placed a stimulating electrode onto the cervical vagus nerve or on its efferent-end and recorded the evoked compound action potential of the three different branches, i.e., apical, middle and main arterial, of the splenic nerve in anesthetized mice (Figure 4A). While all splenic nerve branches were electrophysiologically connected to the vagus nerve, the main arterial splenic nerve showed lower reactivity compared to the two other branches (Figures 4B,C). Interestingly, both the resection of the coeliac/superior mesenteric ganglionic plexus (Figure 4D) or the administration of the α7 ganglionic blocker methyllycaconitine (MLA) (Figure 4E) abolished VNS-induced evoked potential in the middle arterial splenic nerve, demonstrating that the connection between the vagus and splenic nerve occurs through the coeliac ganglion *via* an α7-AChR-dependent pathway. These results are in line with the fact that the CAP is absent in α7-AChR knock-out animals (Wang et al., 2003; Huston et al., 2006; Vida et al., 2011a). Since these results are contributing to the notion that α7-AChR expressed on macrophages could be the main anti-inflammatory mediator in the CAP, the role of α7-AChR expression by macrophages remains to be independently demonstrated using other transgenic models.

**Figure 5:**
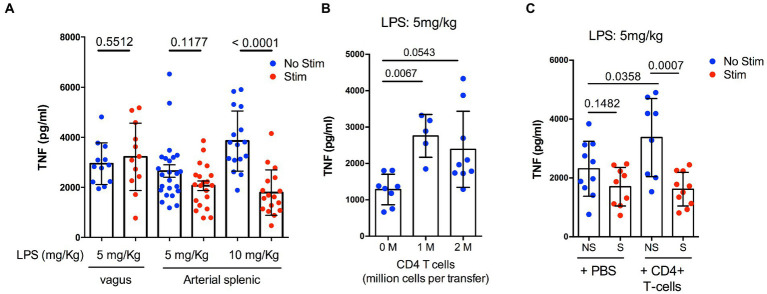
NA promotes TNF suppression *via* β_2_-AR and is independent of T-cells in humans. **(A)** Flow cytometry data showing the expression of β_2_-AR in different populations (CD14^+^ and CD3^+^) of human splenocytes. Secondary antibody alone is shown for each cell types. **(B)** Dose dependent change of LPS-induced TNF caused by treatment of human peripheral blood cells with different concentrations of NA (8 × 10^−5^ – 8 × 10^2^ μM), either in mixed cell culture (all cells) or culture depleted from CD4+ cells. Data are expressed as % over LPS control (*n* = 4). All data are expressed as mean ± SEM.

In this study, we sought to investigate the CAP mechanism in non-irradiated fully conscious animals using the LPS-induced serum TNF secretion gold standard assay, where splenic macrophages are the major source of TNF in blood (Rosas-Ballina et al., 2008). In this model, VNS inhibits TNF secretion in the spleen without affecting TNF production in other organs (Huston et al., 2006; Peña et al., 2011; Vida et al., 2011a). Inasmuch as this pathway is expected to be dependent on α7-AChR (Wang et al., 2003; Huston et al., 2006; Vida et al., 2011a,b), we have tested whether or not the effects of vagus or splenic nerve stimulation can be inhibited by MLA administration. In contrast with VNS, we found that the inhibition of LPS-induced TNF secretion by splenic nerve electrostimulation is not abolished when MLA is administered in mice (Supplementary Figure S1) demonstrating that α7-AChR is not required for splenic nerve stimulation to inhibit inflammation confirming previous reports (Vida et al. a). Since the CAP was also hypothesized to be dependent on ß2-AR expression by CD4^+^ T cells, we tested this hypothesis by using CD4:Cre ADRB2^fl/fl^ mice in which T cells were deficient in β2-AR expression (Supplementary Figure S2). Electrical stimulation of either vagus or splenic nerve inhibited LPS-induced TNF production both in wild type (Figure 5A) and in transgenic (Figure 5B) mice, demonstrating that the CAP is independent of β2-AR expression by T-cells. The CAP is also independent of T-cells expressing Choline AcetylTransferase (ChAT), the enzyme required for acetylcholine synthesis, since CD4:Cre CHATfl/fl mice, in which T-cells were deficient in ChAT expression (Olofsson et al., 2016; Cox et al., 2019; Willemze et al., 2019) still preserved their ability to inhibit LPS-induced TNF secretion upon vagus or splenic nerve stimulation (Figure 5C). Since genetic changes might also give rise to compensatory mechanisms, the role of CD4+ T-cells was assessed in WT mice where 99.6% ±0.23 (*n* = 5) of CD4+ T-cells had been depleted by anti-CD4 antibody treatment (Supplementary Figure S3) at the time of vagus or splenic nerve stimulation. Such treatment did not appreciably impact the CAP providing further evidence that CD4+ T-cells are dispensable (Figure 5D). B-cells have also been suggested to act as intermediary messengers since they represent the majority of splenocytes in the spleen expressing choline acetyltransferase (ChAT), the enzyme required for acetylcholine (ACh) synthesis (Reardon et al., 2013; Hu et al., 2020). To test this hypothesis, we used RAG-1 mice that lack mature T and B cells. The inhibition of LPS-induced TNF production after either vagus or splenic nerve stimulation was comparable in RAG-1^+/+^ and RAG-1^−/−^ mice, demonstrating that functional lymphocytes were not required (Figure 5E). We also found that inhibition of LPS-induced TNF release after VNS was abolished in RAG-1^−/−^ ADRB2^−/−^ mice compared to RAG-1^−/−^ ADRB2^+/+^ mice, further supporting the role of β2-AR signaling on cells other than lymphocytes (Figure 5E).

A direct binding of NA released by splenic nerve to β2-AR on macrophages might explain the anti-inflammatory effect induced by vagus or splenic nerve stimulation (Hu et al., 1991; Chou et al., 1996; Kees et al., 2003). To test this hypothesis, we took advantage of the LysM:Cre ADRB2^fl/fl^ mice in which β2-AR alleles are deleted in the myeloid lineage cells (Matheis et al., 2020; Wieduwild et al., 2020). We found that the inhibition of LPS-induced serum TNF levels is almost completely abolished when β2-AR are absent from the surface of myeloid cells demonstrating that NA is directly binding to β2-AR on myeloid cells (Figure 5F).

Some results reported in the literature using FoxN1^−/−^ (nude) mice are in contradiction with our conclusion. As other authors (Peña et al., 2011; Vida et al., 2011b; Olofsson et al., 2012), we (Guyot et al., 2019) found that the CAP was abolished in nude mice when using a 5 mg/kg LPS dose (Figure 1A). Similar results were obtained when the splenic nerve was stimulated (Figure 1A). We tested different hypotheses that might explain the discrepancy with our previous results showing that lymphocytes are dispensable to the CAP. This discrepancy could be explained by lower density innervation of the spleen, by a difference in β2-AR signaling or by a lower sensitivity of nude mice to LPS challenge. The examination of whole spleen staining for tyrosine hydroxylase (TH) by light sheet microscopy did not reveal difference between splenic adrenergic innervation of FoxN1^+/+^ and FoxN1^−/−^ mice (Supplementary Figure S4A). To test whether β2-AR signaling might be different between FoxN1^+/+^ and FoxN1^−/−^ mice, sorted splenic macrophages were stimulated *in vitro* with LPS and incubated in the presence of different concentrations of a β2-AR agonist (salbutamol). No significant difference was found between TNF levels in supernatants from wild type and FoxN1^−/−^ mice demonstrating that nude macrophages can be inhibited by β2-AR engagement (Supplementary Figure S4B). We also found that LPS-induced TNF secretion can be inhibited *in vivo* by administration of salbutamol in FoxN1^−/−^ mice demonstrating that nude mice retain their intrinsic ability to inhibit inflammation when β2-AR signaling is engaged (Supplementary Figure S4C). Interestingly, when nude mice were challenged with a higher dose of LPS (10 mg/kg) a CAP was observed, demonstrating that this pathway is indeed functional in nude mice (Figure 2A). The absence of CAP previously observed in nude mice (Peña et al., 2011; Vida et al., 2011b; Olofsson et al., 2012; Guyot et al., 2019) might be explained by the low-dose (<6 mg/kg) of LPS challenge used in most of these studies including ours. We also challenged results of CD4^+^ T-cell transfer experiments in nude mice (Rosas-Ballina et al., 2011; Vida et al., 2011b; Olofsson et al., 2012) and found that such CD4^+^ T-cell transfer led to the increase of LPS-induced serum TNF levels *per se* (Figure 1B). This increased LPS-induced TNF levels by CD4^+^ T cell transfer allow the CAP to be revealed when the splenic nerve is electrostimulation (Figure 1C). Altogether, our results demonstrated that CAP is indeed functional in nude mice.

In order to assess whether CD4^+^ T cells are dispensable for CAP in translational models, we performed additional experiments using pig and human experimental platforms. The pig represents a good model of the human splenic neuroanatomy. Spleens from both species are innervated exclusively by peri-arterial nerves that carry sympathetic (TH^+^) fibres with no cholinergic innervation observed (Heusermann and Stutte, 1977; Verlinden et al., 2019). To evaluate the modulatory effects of NA on pig and human splenocytes, we measured the effects of increasing concentrations of NA on LPS-induced TNF production. NA was able to suppress TNF in a dose-dependent manner in both species (Figure 2A). To assess which neurotransmitter receptor was responsible for the observed effect, we selected a dose of NA (8 μM) able to significantly suppress TNF in both species and added increasing concentrations of various receptor antagonists. The blockade of α-AR with phentolamine (Phe) did not cause a loss of TNF suppression compared to LPS plus NA only (Figure 2B for % over LPS and Supplementary Figure S5A for raw data). In contrast, blocking β-AR with propranolol (Pr) resulted in a clear loss of inhibitory effect in both species (Figure 2C for % over LPS and Supplementary Figure S5B for raw data). To investigate the possible role of ACh, we blocked either all the nicotinic AchR (ɑ-bungarotoxin or Hexamethonium) or specifically the α7-AchR (MLA) using different antagonists. No loss of LPS-induced TNF inhibition was observed in either species with any of the antagonists (for MLA: Figure 2D for % over LPS and Supplementary Figure S5C for raw data; for α-bungarotoxin and hexamethonium Supplementary Figure S6) demonstrating the absence of role for nicotinic AchR in the β-AR-mediated inhibition of TNF production.

We then evaluated the expression of β-AR on different cell populations within human isolated splenocytes by staining for β_2_-AR. The receptor was found to be expressed by the vast majority of CD14^+^ cells and in a smaller proportion of T-cells (Figure 3A). Finally, we depleted CD4+ T-cells by magnetic sorting from either peripheral blood mononuclear cells (PBMCs) (Figure 3B, *N* = 4, for % over LPS and Supplementary Figure S7 for raw data) or splenocytes (Supplementary Figure S8, *N* = 3) and assessed the dose response to NA compared to the original mixed culture. We detected no difference between the dose response when CD4+ T cells were removed from the culture.

## Discussion

For a long time, a connection between VN and the SpN has been speculated. Only recently Gonzalez-Gonzalez et al. have addressed the question of electrophysiological connection between the vagus and the splenic nerve in rats (Gonzalez-Gonzalez et al., 2021). They found that VNS increases firing rate in some of the splenic nerve branches, opening the possibility of a direct or indirect connection between the splenic and that the vagus nerve. To address this question we have adopted a more direct approach by quantifying evoked compound action potential following VNS and by exhaustively testing all arterial and non-arterial branches connecting the spleen to the body. We found that, while all splenic nerve branches were electrophysiologically connected to the vagus nerve, the main arterial splenic nerve showed lower reactivity compared to the two other branches. This result is in line with the variable density of axons connecting the VN to the spleen found in the different arterial splenic nerve branches by viral tract tracing (from 41% to <5%) (Gonzalez-Gonzalez et al., 2021). This observation might also explain why Bratton et al. could not find any impact of vagal efferent stimulation on splenic nerve activity in rats (Bratton et al., 2012).

This electrophysiological study also reveals that the connection between the vagus and splenic nerve occurs through the coeliac ganglion *via* an α7-AChR-dependent pathway. This neural connection was initially proposed by Vida et al. (2011a) but later refuted (Olofsson et al., 2012). The fact that the CAP was not observed in α7-AChR knock-out animals (Wang et al., 2003; Huston et al., 2006; Vida et al., 2011a) and that nicotine exerts anti-inflammatory effects on macrophages *via* α7-AChR (Borovikova et al., 2000; Wang et al., 2003; Uni et al., 2020) led to the notion that α7-AChR expressed on macrophages is the main anti-inflammatory mediator of vagus nerve neuromodulation. However, even if the impact of AChR agonists-antagonists administration on pro-inflammatory cytokine secretion by macrophages is not in doubt, the dependency of the CAP on α7-AChR expression by macrophages in physiological condition needs to be addressed.

Since all studies exploring CAP mechanism were obtained by performing electrostimulation within a few hours of anaesthesia (Huston et al., 2006; Rosas-Ballina et al., 2008, 2011; Peña et al., 2011; Vida et al., 2011a,b; Olofsson et al., 2012), in immunodeficient animals (Peña et al., 2011; Rosas-Ballina et al., 2011; Vida et al., 2011b; Olofsson et al., 2012; Guyot et al., 2019) or in irradiated-reconstituted animals (Olofsson et al., 2012), we have investigated the CAP mechanism in non-irradiated fully awake animals. Using multiple transgenic models, we found that CD4+ cells are dispensable to the CAP whether it is triggered by vagus or splenic nerve stimulation. Since genetic changes might be associated with compensatory mechanisms, we have confirmed that CD4^+^ T-cells are not necessary for the inhibition of TNF release by splenic or vagus nerve electrostimulation in CD4-depleted mice. These results seem at odds with those reported by Pena et al. who found that the CAP was inhibited by anti-CD3ε depleting antibody (Peña et al., 2011). However, the expression of CD3ε by peripheral nerves (Galbavy et al., 2015) might explain this discrepancy due to antibody-mediated destruction of post-ganglionic adrenergic fibers.

A retrospective analysis of the data in the literature shows that the role of CD4+ T-cells in the CAP was mainly supported by experiments showing the absence of CAP in nude (FoxN1^−/−^) mice (Peña et al., 2011; Rosas-Ballina et al., 2011; Vida et al., 2011b; Olofsson et al., 2012; Guyot et al., 2019) and the restoration of CAP when these mice were transferred with WT CD4^+^ T cells (Rosas-Ballina et al., 2011; Vida et al., 2011b; Olofsson et al., 2012). Our data suggest that the CAP effect is not apparent in nude mice when using low doses of LPS but is present and active when using high doses of LPS. Moreover, we have noticed that CD4^+^ T cell transfer to nude mice led to an increase of LPS-induced TNF levels and restoration of the capacity of splenic nerve electrostimulation to inhibit inflammation. Since all data reporting transfer into nude mice in the literature are expressed as percentage relative to sham non-transferred animals (Rosas-Ballina et al., 2011; Vida et al., 2011b; Olofsson et al., 2012), it is unfortunately not possible to evaluate the impact of CD4^+^ T cell transfer on LPS-induced serum TNF production. However, while no statistical analyses were provided, absolute serum TNF values are reported in two of these publications (Rosas-Ballina et al., 2011; Olofsson et al., 2012), which support our finding that CD4^+^ T cell transfer increases LPS-induced TNF production. A potential explanation for this intriguing finding is the alterations in the levels of catecholamine-degrading enzymes in the spleen after an inflammatory stimulus (Monteiro et al., 2020; Ostadkarampour and Putnins, 2021). It is possible that such effects are associated with alterations in the levels of noradrenaline-degrading enzymes in Nude mice during LPS 10 mg/kg that is not observed at 5 mg/kg. Altogether, our results demonstrated that CAP is functional in nude mice thereby challenging this evidence as a demonstration of the role of CD4^+^ T-cell in the vagus nerve anti-inflammatory pathway.

These results have some implications for our understanding of the control of immune responses in pathological conditions. It was shown that, in experimental models of infection like sepsis, β2-AR antagonist administration increases susceptibility to LPS-induced endotoxemia (Grailer et al., 2014) and ADRB2 knockout animals were highly susceptible to LPS-mediated endotoxemia (Ağaç et al., 2018). Whether this action is due to a direct role for catecholamines in suppressing inflammation was not known. Our study as well as previous reports (Ağaç et al., 2018) show that selective deletion of ADRB2 on innate immune cells increases inflammation suggesting an intrinsic role for catecholamines in suppressing inflammation, which is distinct from the indirect actions of ACh.

**Figure 6:**
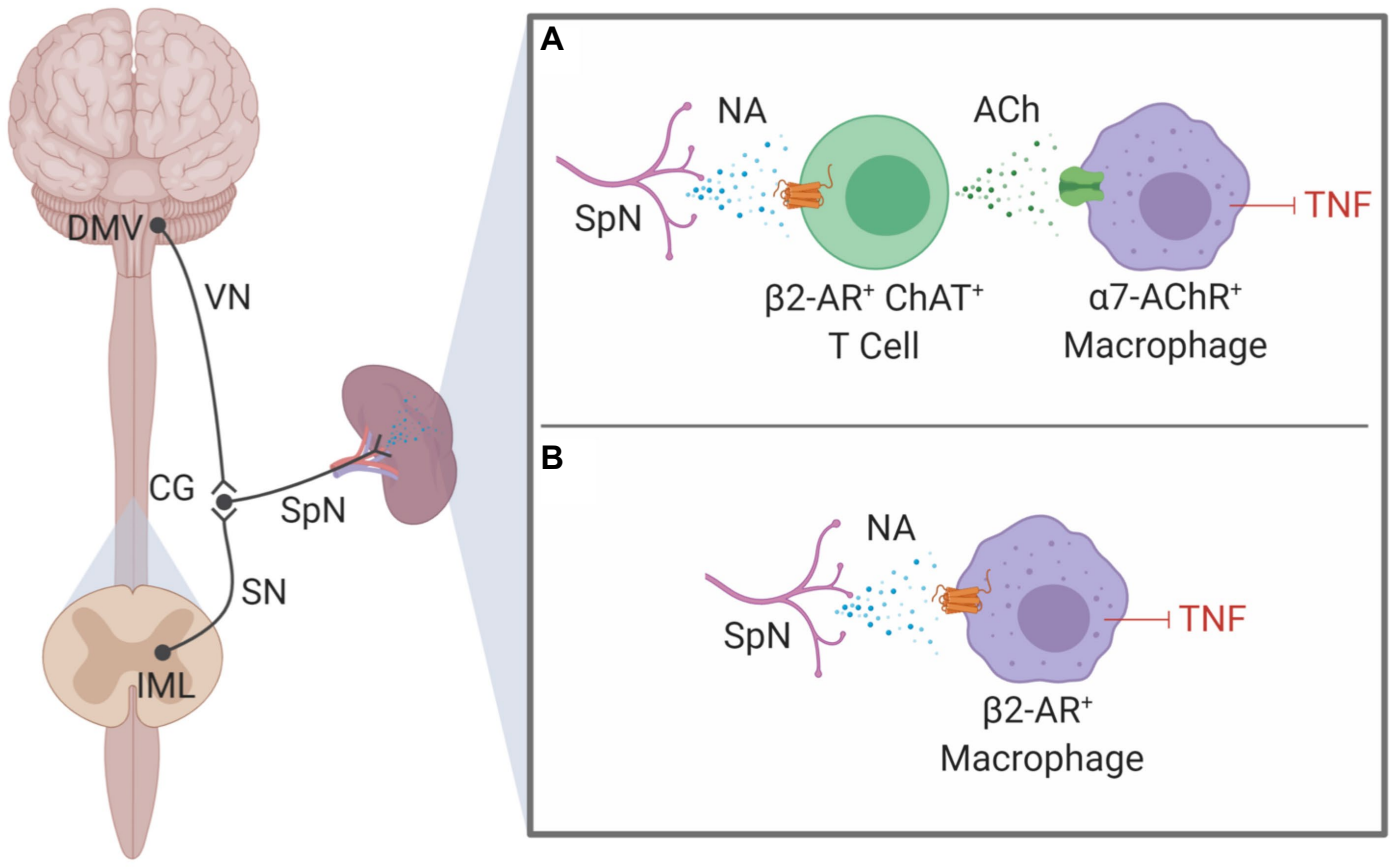
Cholinergic anti-inflammatory pathway revisited. The efferent arm of the inflammatory reflex, is composed of the vagus and the splanchnic nerve (SN) originating from the dorsal motor nucleus (DMV) and the intermediolateral nucleus (IML) respectively. Both nerve converge to the coeliac ganglion (CG) where they release acetylcholine (ACh) that binds to the α7 subunit of the nicotinic acetylcholine receptor (α7-AChR) relaying the neural information to the adrenergic splenic nerve (SpN). **(A)** In the previously described CAP (Gallowitsch-Puerta and Pavlov, 2007; Jonge and Ulloa, 2009; Andersson and Tracey, 2012; Olofsson et al., 2012), the noradrenaline (NA) release at the splenic nerve termini, which binds to β2-adrenergic receptor (β2-AR) of CD4+ T-cells, which in turn triggers the release of ACh. Engagement of the α7-AChR at the surface of macrophage by locally produced ACh inhibits their production of TNF following LPS exposition. **(B)** Our data support a simpler model where, NA binds directly to β2-AR to inhibit LPS-induced TNF secretion.

In conclusion, while the impact of AChR agonist administration on pro-inflammatory cytokine secretion by macrophages *via* α7-AChR is without question, we found that the activation of the CAP by stimulation of vagus or splenic nerves is not dependent on CD4^+^ T-cells as previously reported but rather on direct action of NA on β2-AR on splenic macrophages (Figure 6).

## Data availability statement

The raw data supporting the conclusions of this article will be made available by the authors, without undue reservation.

## Ethics statement

The studies involving human participants were reviewed and approved by University of Cambridge ethical committee (REC Ref: 15/EE/0152). The patients/participants provided their written informed consent to participate in this study. The animal study was reviewed and approved by Comité Institutionnel d’Éthique Pour l’Animal de Laboratoire (CIEPAL) and the Royal Veterinary College Animal Welfare.

## Author contributions

PB conceived the study. TS and PB designed mice related experiments. TS, CP, JL, MG, NH, EM, and SH performed mice related experiments and analysis. MD, JK, and JP designed pig related experiments. MD and JK performed related pig experiments MD, ND, and KS-P designed human related experiments. ND performed human related experiments. TS, MD, ND, JK, AS, MV, NG, and PB interpreted the data. PB wrote the manuscript. All authors contributed to the article and approved the submitted version.

## Funding

The work was supported by the CNRS, Galvani Bioelectronics and the Agence National de la Recherche (ANR) grant #ANR-21-CE18-0016.

## Conflict of interest

The authors declare that the research was conducted in the absence of any commercial or financial relationships that could be construed as a potential conflict of interest.

## Publisher’s note

All claims expressed in this article are solely those of the authors and do not necessarily represent those of their affiliated organizations, or those of the publisher, the editors and the reviewers. Any product that may be evaluated in this article, or claim that may be made by its manufacturer, is not guaranteed or endorsed by the publisher.
