# Supplementary material for: The cholinergic anti-inflammatory pathway inhibits inflammation without lymphocyte relay
Source: Front Neurosci. 2023 Apr 14;17:1125492. doi: 10.3389/fnins.2023.1125492 (PMC10140439; doi:10.3389/fnins.2023.1125492)
Supplement: Supplementary file 1 [file Presentation_1.pdf]

SUPPLEMENTAL MATERIALS AND METHODS

Chemicals

α-Bungarotoxin (cat. no. 2133, reconstituted with ddH_2O) and Hexamethonium bromide (cat. no. 4111, reconstituted with ddH_2O) were purchased from Tocris Bioscience.

Quantification of β2-adrenergic receptor at the surface of CD4 T cells. CD4 T cells were isolated from splenocyte population using EasySep™ Mouse T Cell Isolation Kit (Stemcell) according to manufacturer's instructions. Purity of the isolated CD4 T lymphocytes was determined to be over 95% using flow cytometry. CD4 T cells were incubated with 10 nM of a tritiated β1/β2-adrenergic receptor antagonist (4-[3-[(1,1-Dimethylethyl)amino]2-hydroxypropoxy]-1,3-dihydro-2H-benzimidazol-2-one hydrochloride, also known as CGP-12177) for 4 hours at 4˚C. After incubation with $^{[3H]}$CGP-12177, cells were washed four times with ice-cold PBS and lysed in PBS, 0.1% Triton X-100, 2.5 mM EDTA. Radioactivity was measured using a scintillation liquid analyzer and expressed as count per minutes (cpm) per million cells.

Incubation protocols for human and pig splenocytes

LPS (Sigma Life Sciences, cat. no. L4391) and L-Norepinephrine hydrochloride (Sigma Life Sciences, cat. no. 74480) were reconstituted in saline at 1 mg/mL and 10 mg/mL, respectively, and aliquots stored at -20 °C. α-Bungarotoxin (cat. no. 2133) was reconstituted with ddH_2O at 1mM, and Hexamethonium bromide (cat. no. 4111) were reconstituted with ddH_2O at 100mM. All compounds were stored at -20 °C then thawed at room temperature and added to cells 30 minutes before LPS and/or NA at final concentrations of 0.15-15 µg/mL (α-Bungarotoxin) and 0.1-100 µM (Hexamethonium). Aliquots of NA and LPS were thawed at room temperature, and LPS sonicated for 5 minutes. Compounds were diluted in culture medium as required, vortexing for 10 seconds between each serial dilution for dose-response experiments.

Magnetic separation of mouse splenic macrophages

F4/80+ macrophages were isolated from splenocyte population using F4/80-PE antibody (clone BM8, thermofisher) and EasySep™ Mouse PE Positive Selection Kit II (Stemcell) according to

manufacturer's instructions. Purity of the isolated macrophages was checked using flow cytometry.

Light sheet

Light-sheet-based fluorescent microscopy: Animals were perfused using a 4% PFA solution followed by a PBS solution. Excised spleens were depigmented and clarified using the iDisco+ method (https://idisco.info/). Briefly, they were dehydrated at room temperature in successive bathes of 20% MetOH for 1h, 40% MetOH for 1h, 60% MetOH for 1h, 80% MetOH for 1h, 100% MetOH for 1h and 100% MetOH overnight. Then the organs are incubated in a solution of 33% MetOH and 66% Di-ChloroMethan (DCM, Sigma) overnight and washed twice with 100% methanol for 1h. Organs were then bleached in chilled fresh 5% H202 in methanol overnight at 4°C before being rehydrate with methanol/H2O series (80%, 60%, 40%, 20% and PBS, 1 h each at RT). Samples were them immunolabelled for 24 h with anti-TH (AB152, Merck) after overnight permeabilization at 37°C and overnight blocking in blocking solution (1,7% TritonX- 100, 6% donkey serum and 10% DMSO in PBS). After 3 washes in PBS/ 0.2%Tween-20, samples were incubated with the secondary antibody (donkey anti-goat from Jackson Immunoresearch) for 24h. Samples were then dehydrated at room temperature in successive bathes of 20% MetOH for 1h, 40% MetOH for 1h, 60% MetOH for 1h, 80% MetOH for 1h, 100% MetOH for 1h and 100% MetOH overnight. Then the organs are incubated in a solution of 33% MetOH and 66% Di-ChloroMethan (DCM, Sigma) for 3h at RT, then in 100% DCM twice for 15 min twice and transferred overnight into the clearing medium 100% DiBenzylEther 98% (DBE, Sigma). Imaging was performed using a home-made light-sheet ultramacroscope. The specimen was placed into a cubic cuvette filed with DBE placed on the Z-stage of the bench. It was illuminated with planar sheets of light, formed by cylinder lenses. The light coming from a multi-wavelength (561 nm) laser bench (LBX-4C, Oxxius) was coupled via two single mode optical fibers into the setup, allowing illumination from one or two sides. We used two-sided illumination. The specimen was imaged from above with a MVX10 macroscope, through a PlanApo 2X/0.5 NA objective (Olympus) with an additional zoom of the macroscope of 1.6, which was oriented perpendicular to the 561nm light sheet. Images were captured using a sCMOS Camera (Orca-Flash4.0) synchronized with the z-stage moving the sample through the light sheet. The ultramicroscope is managed by Micro-manager software and z-stacks of images were taken every 2 µm. The images

stacks are fused using the alpha-blending method with a home-made ImageJ macro (Rasband, W.S., ImageJ, U. S. National Institutes of Health, Bethesda, Maryland, USA, http://imagej.nih.gov/ij/, 1997e2012).

SUPPLEMENTAL FIGURES

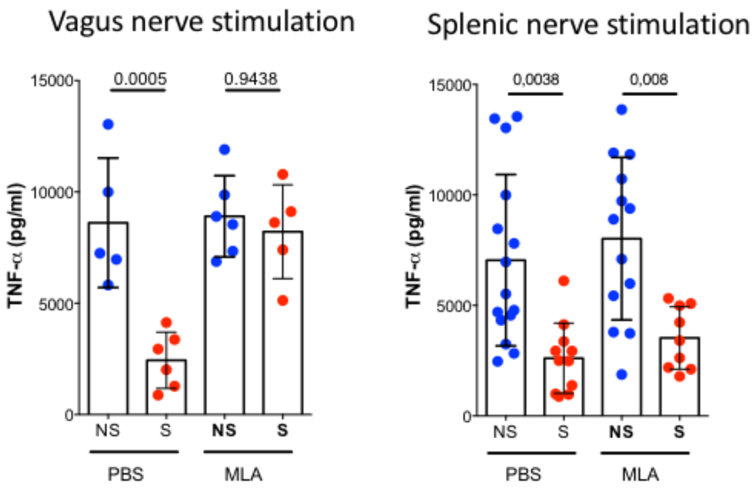

Supplemental Figure 1. Wild type mice were implanted onto the vagus or main arterial splenic nerve with a micro-cuff electrode. One week after surgery, LPS (5 mg/kg) was injected and electrical stimulation was applied or not (650 μA, 10 Hz, 2 min duration, -20, 0 and +10 relative to LPS injection) to the vagus (left panel) or main arterial splenic nerve (right panel) in freely moving animals. Data show serum TNF levels in individual mice +/- S.E.M. of 2 experiments in non-electrically stimulated (NS, blue) and electrically stimulated (S, red) mice. P-value of Mann-Whitney test are reported.

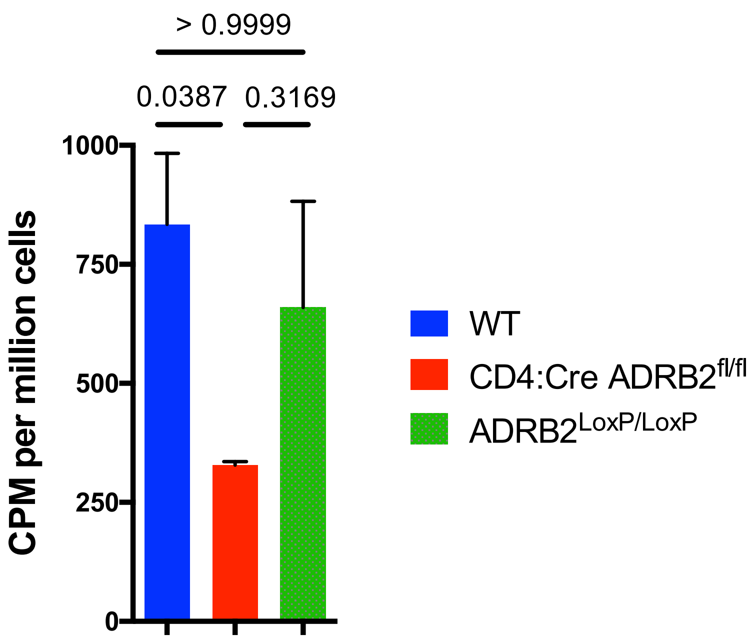

Supplemental Figure 2. Loss of β2-AR expression on CD4 T-cell in CD4:Cre ADRB2$^{fl/fl}$ mice. CD4 T cells from wild type, CD4:Cre ADRB2$^{fl/fl}$, ADRB2$^{LoxP/LoxP}$ mice were sorted using anti-CD4 magnetic beads. After incubation with radioactive β1/β2-AR antagonist ($^{[3H]}$CGP-12177), cell membrane radioactivity was measured. Results are expressed as mean count per minutes (CPM) per million CD4 T cells +/- S.E.M. of 2 experiments.

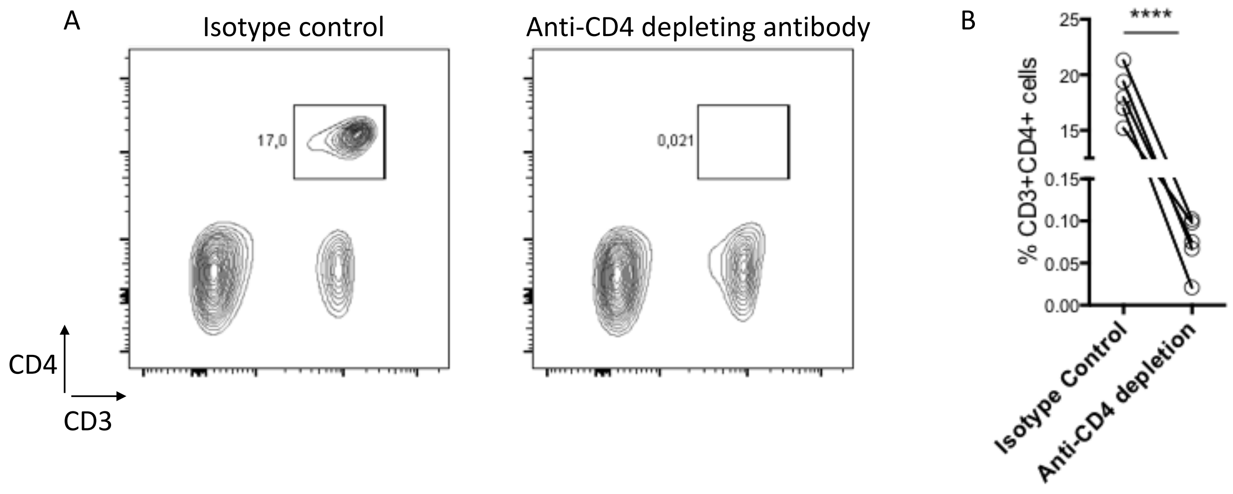

Supplemental Figure 3. Depletion of CD4 T cell after administration of anti-CD4 depleting antibody. Wild type mice were i.p. injected with CD4 depleting antibody or isotype control three days and one day before harvesting splenocytes. A flow cytometry analysis was performed after staining with anti-CD3 and anti-CD4 antibodies. A flow cytometry profile from one presentative animal (A) and quantification of CD4 depletion in five mice (B) are presented.

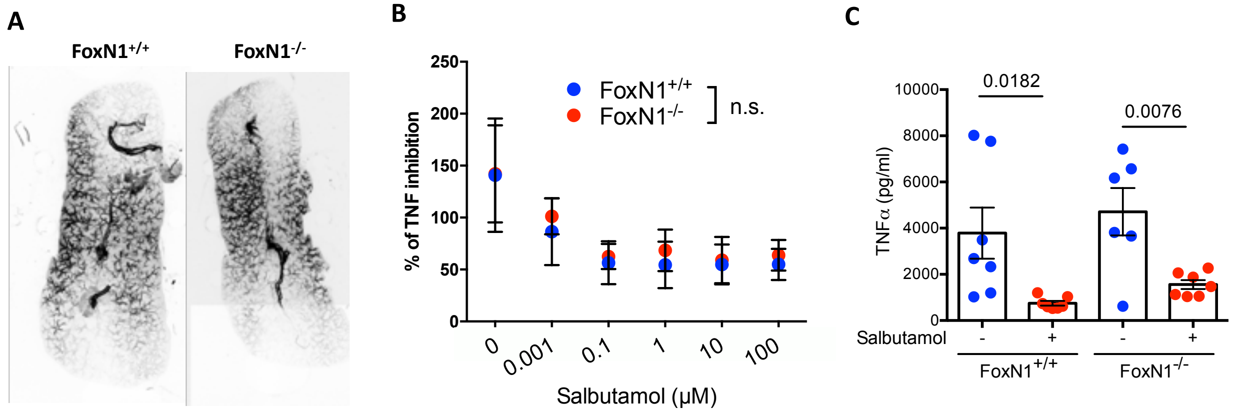

Supplemental Figure 4. Salbutamol-mediated inhibition of LPS-induced TNF release in wild type and FoxN1$^{-/-}$ (nude) mice. (**A**) Representative light sheet imaging of whole spleen of wild type and FoxN1$^{-/-}$ mice after staining for TH (n = 3/group). (**B**) F4/80+ sorted splenocytes were stimulated *in vitro* with LPS (100 ng/mL) and were incubated in the presence of different concentrations of salbutamol. Sixteen hours later TNF was measured in the supernatants. No significant difference was found between TNF levels in supernatants from wild type and FoxN1$^{-/-}$ mice using regular two-way ANOVA followed by Sidak's multiple comparison test. One representative experiment is shown out of 3. (**C**) LPS (5 mg/kg) was injected together (+) or not (-) with Salbutamol (20 mg/kg) to wild type and FoxN1$^{-/-}$ mice. Data show serum TNF levels in individual mice +/- S.E.M. of 2 experiments. t-test were performed.

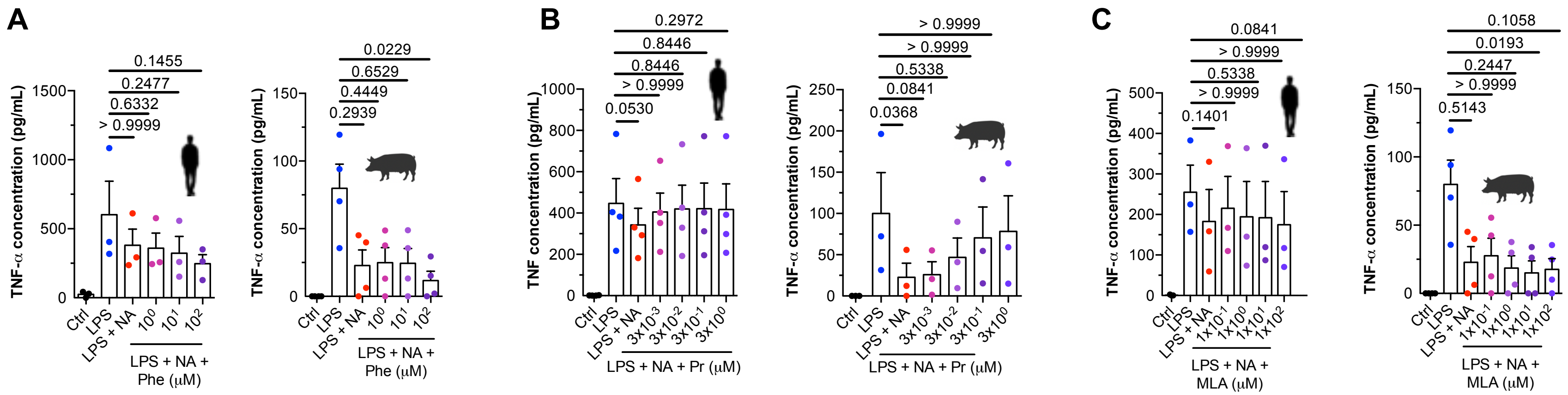

Supplemental Figure 5. NA promotes TNF suppression via β-AR and is independent of α-AR in humans and pigs. (**A-D**) Quantification of TNF in human and pig splenocytes in medium only (Ctrl), LPS (100 ng/mL), LPS (100 ng/mL) + NA (8 μM) and LPS (100 ng/mL) + NA (8 μM) in the presence of various concentrations of α-AR (Phentolamine, Phe) (**A, B**), β-AR (Propranolol, Pr) (**C**) or α7-AChR (methyllycaconitine, MLA) (**D**) receptor antagonists. Data are either expressed as % over LPS control (A) or as TNF concentration in pg/mL (B-D). All data are expressed as mean ± SEM.

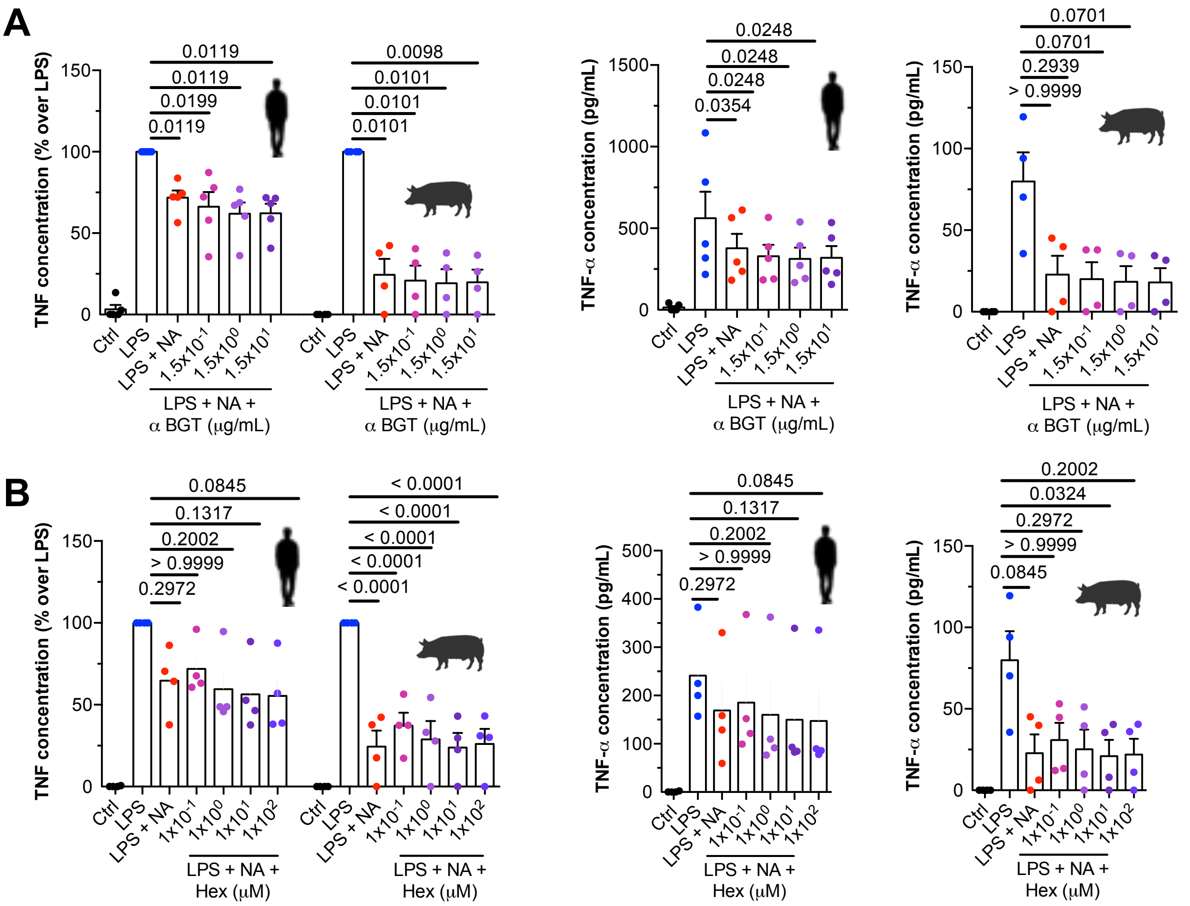

Supplemental Figure 6. NA promotes TNF suppression independently of α7-AChR in humans and pigs. (**A, B**) Quantification of TNF in human and pig splenocytes in medium only (Ctrl), LPS (100 ng/mL), LPS (100 ng/mL) + NA (8 μM) and LPS (100 ng/mL) + NA (8 μM) in the presence of various concentrations of α-bungarotoxin (α-BG), or hexamethonium (Hex). Data are expressed as mean ± SEM either of % over LPS control or of TNF concentration in pg/mL.

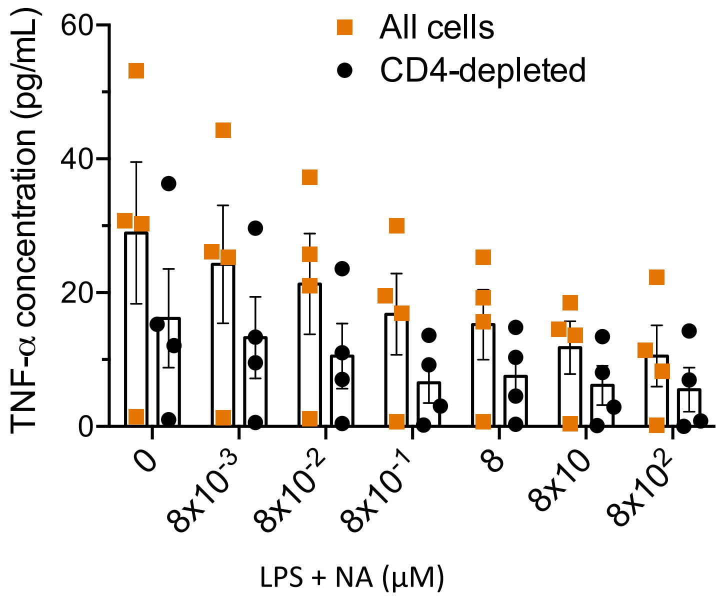

Supplemental Figure 7. NA inhibit LPS-induced TNF secretion in human PBMCs culture before or after CD4 T-cell depletion. Mean TNF concentration in pg/mL (± SEM, n = 4 donors) in total human PBMCs (All cells) or after magnetic depletion of CD4 T-cell (CD4-depleted) after addition of LPS alone (0 μM) or LPS (100 ng/mL) with various concentration of NA.

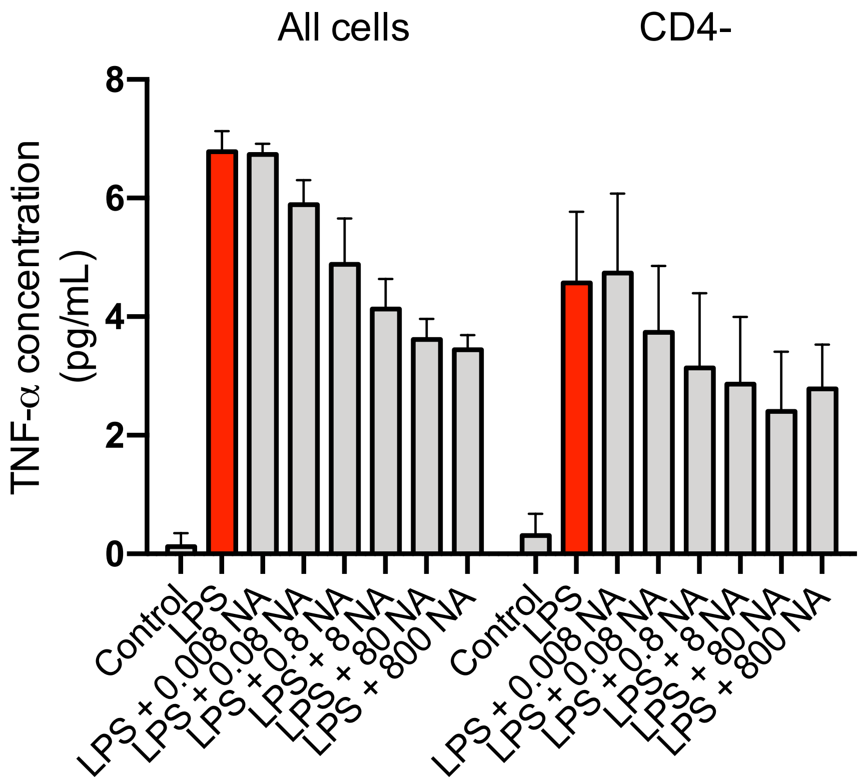

Supplemental Figure 8. NA promotes TNF suppression in human splenocytes. Quantification of TNF in total human splenocytes (All cells) or after magnetic depletion of CD4 T-cell (CD4-depleted) after addition of LPS alone (0 μM) or LPS (100 ng/mL) + NA (8 μM). Data are expressed as mean TNF concentration in pg/mL (± SEM, N = 3 donors).
